# Phagocytosis of Gut Bacteria by *Entamoeba histolytica*

**DOI:** 10.3389/fcimb.2019.00034

**Published:** 2019-02-26

**Authors:** Lakshmi Rani Iyer, Anil Kumar Verma, Jaishree Paul, Alok Bhattacharya

**Affiliations:** School of Life Sciences, Jawaharlal Nehru University, New Delhi, India

**Keywords:** microbiota, phagocytosis, *Entamoeba histolytica*, metagenomics, *Lactobacillus ruminus*

## Abstract

The protist parasite *Entamoeba histolytica* causes amoebiasis, a major public health problem in developing countries. Only a small fraction of patients infected with the parasite display invasive disease involving colon or extra intestinal tissues such as liver. *E. histolytica* exists as two distinct forms, cysts, the infective form, and trophozoites, that are responsible for disease pathology. The latter multiply in the large intestine occasionally causing disease. The large intestine in humans is populated by a number of different bacterial communities and amoebic cells grow in their midst using some as food material. Several studies have shown relationship between bacteria and *E. histolytica* growth and virulence. However, an understanding of this relationship in human gut environment is not clear. We have investigated the possibility that there may be specific interaction of amoeba with different bacteria present in the gut environment by using a metagenomic pipe line. This was done by incubating bacteria isolated from human fecal material with *E. histolytica* and then identifying the bacterial population isolated from amoebic cells using a rRNA based metagenomic approach. Our results show that the parasite prefers a few bacterial species. One of these species is *Lactobacillus ruminus* which has never shown to be associated with *E. histolytica*.

## Introduction

Amoebiasis is caused by the protist parasite *Entamoeba histolytica* affecting both intestinal and extra intestinal tissues. It is the third leading cause of death from parasitic diseases worldwide, mainly in developing countries. It is estimated that approximately 50 million people worldwide are infected with this parasite, resulting in 40–100 thousand deaths annually (Haque et al., 2003). In the human gut, *E. histolytica* cells reside and multiply in the presence of a large variety of microorganisms. *E. histolytica* consumes bacteria by phagocytosis. Phagocytosis is an essential process in this organism and blocking this process leads to inhibition of proliferation and loss of pathogenicity of amoebic cells (Hirata et al., 2007). It is therefore believed that the interaction between bacteria and amoeba plays an important role in the growth and virulence of the parasite (Sahoo et al., 2004)

The human gut is made up of a complex community of hundreds of species of microbes, mostly commensal in nature. A high biodiversity of the gut microbiota is associated with a healthy state. Microbiota compositions can vary significantly from person to person, even within healthy individuals or twins in the same household (Lozupone et al., 2012; Smith et al., 2013). Several studies have noted that the bacterial microbiota may influence the behavior and virulence of individual pathogens, their immune response, and lead to variability in the outcome of parasitic infections (Marie and Petri, 2014; Bär et al., 2015). These organisms form a symbiotic relationship that influences human physiology and disease progression (Lozupone et al., 2012; Sassone-Corsi and Raffatellu, 2015). Metagenomic studies on gut microbiome have generated a wealth of information, that has enabled a critical examination of the fluctuations in the gut microbiome during different disease conditions for any possible medical intervention.

A few studies have revealed that infection with *E. histolytica* is significantly correlated with fecal microbiome composition and diversity. Coculture with *Escherichia coli* can augment the virulence of an amoebic cell line (Padilla-Vaca et al., 1999). Epithelial monolayers exposed to enteropathogenic bacteria become more susceptible to damage by *E. histolytica*. Varet et al. (2018) have shown that live bacteria of the family *Entereobacteriaceae* help *E. histolytica* to survive oxidative stress and establish itself in the intestinal mucosa by causing changes in genes involved in glycolysis and proteasome activity. However, these changes did not occur in the presence of *Lactobacillus acidophilus* which could be explained by the fact that the probiotic effect of bacteria such as *L. acidophilus is* mediated by the ability to produce H_2_O_2_ and to maintain a normal, homeostatic microbiota. Further it was shown that phagocytosis of pathogenic *E. coli* by amoebae increased epithelial cell damage (Galván-Moroyoqui et al., 2008). Two separate studies showed that individuals with symptomatic infection due to *E. histolytica* correlated with the variable levels of members belonging to the *Prevotellaceae* family. A cohort study conducted in south west Cameroon showed that the outcome of amoeba infections can be predicted accurately from the composition of an individual's gut microbiota, and members of *Prevotellaceae* was found to be one of the most prevailing taxa in individuals with asymptomatic infection whereas *Prevotella copri* and *Prevotella stercorea* are found to be downregulated in infected individuals (Morton et al., 2015). In another study conducted in children with diarrhea in Bangladesh, elevated levels of *P. copri* were found in patients with diarrheagenic *E. histolytica* infections suggesting the influence of the microbiome on the state of the gut (Gilchrist et al., 2016). Elevated levels of *P. copri* are also associated with risk of autoimmune disease, colitis and inflammation suggesting that inflammation driven by the gut microbiome has the potential to influence the outcome of an infection (Scher et al., 2013). A study on intestinal parasite infection in individuals from southern Côte d'Ivoire demonstrated a significant increase in the relative abundance of *Bifidobacterium* in *Giardia duodenalis* positive patients. This study suggested that intestinal protists such as *E. histolytica, G. duodenalis*, and *Blastocystis hominus* can induce significant changes in the intestinal microbiome that result in substantially different bacterial communities (Burgess et al., 2017). Real time analysis on the gut flora of patients infected with *E. histolytica* from our laboratory have shown significant changes in the indigenous gut flora of infected individuals showing increase in abundance of Bifidobacterium and depletion of a few predominant genera in the gut like *Bacteroides, Lactobacillus, Clostridium leptum, Clostridium coccoides* subgroup*, Campylobacter*, and *Eubacterium*. It was concluded that the decrease in beneficial bacterial population leads to dysbiosis of gut bacteria influencing the outcome of disease (Verma et al., 2012).

In the present study we have investigated the possibility that *E. histolytica* may prefer specific bacteria for phagocytosis in colonic lumen. We have identified the bacteria that are preferentially taken up by amoeba from total bacterial population prepared from human fecal material using rRNA based metagenomic analysis.

## Methodology

### Culture of *E. histolytica* and Bacterial Cells

Axenic strain of *E. histolytica*, HM-1:IMSS was cultured in TYI-S-33 medium (Clark and Diamond, 2002), in 250 ml culture flasks for 72 h at 35.5°C. The spent medium was discarded and 5 ml of fresh prewarmed culture medium was added to the flask. The walls of the flasks were tapped to detach the trophozoites followed by incubation of the flasks in ice water for 5 min. The trophozoites were harvested by pelleting at 300 g for 5 min at 25°C. The pelleted cells were resuspended in incomplete medium (TYI-S-33 medium without adding serum), number of cells were counted using a haemocytometer and then placed at 35.5°C till used for incubation with bacteria (1 amoeba: 1000 bacterial cells).

*E. coli* cells (O55 and C41-DE3) were incubated in LB medium at 37°C for 12–16 h and harvested by centrifugation at 14,000 g. The cells were then washed with Phosphate-buffered Saline (PBS) maintained at 7.4 pH, twice to remove the medium before use. The bacterial cells were counted by measuring the O.D at 600 nm.

### Uptake of Bacteria

The O55 *E.coli* cells were incubated with the trophozoites in incomplete TYI-S-33 medium at 37°C in the ratio of 1:1000 for indicated time periods to allow phagocytosis to take place. The unphagocytosed bacteria were removed by washing twice with ice cold PBS containing 5 mM Na azide and 50 uM gentamycin at 300 × g for 5 min at 25°C to remove the non-phagocytosed bacteria sticking to the surface of the parasite and finally with PBS to remove traces of the antibiotic. The absence of bacteria sticking to the surface was checked by plating an aliquot of the washed trophozoites before performing the lysis step following the protocol of (Galván-Moroyoqui et al., 2008). The trophozoites were lysed using 100 μl of 0.12% Triton X-100 in LB medium for 3 min. The lysate was plated onto LB agar plates after serial dilution and incubated overnight at 37°C. Colonies were counted and the optimum time necessary for maximum phagocytosis to occur was inferred from the result. As a negative control, the bacteria were incubated with the trophozoites at 4°C and plated under the same conditions.

### Assay of Phagocytosis of *E.coli* Cells by Fluorescent Microscopy

In order to visualize the phagocytosis of bacteria by the parasite, GFP expressing recombinant *E.coli* cells (C41-DE3) were incubated with *E. histolytica* (HM-1:IMSS) cells in the ratio 1:1,000 for different time periods at 37°C in 200 μl of incomplete TYI-S-33 medium. After incubation, the amoebae were pelleted by centrifugation at 300 × g for 5 min at 25°C. The unphagocytosed bacteria adhering to the outer surface of amoeba were removed by washing in 500 μl PBS containing 5 mM Na Azide and 50 μM gentamycin at 300 × g for 5 min at 25°C twice and finally with PBS once. Amoebic cells were finally suspended in 1 ml PBS. The cells were transferred to acetone cleaned coverslips placed in a prewarmed petridish and placed at 35.5°C for 5–10 min. PBS was discarded, and the cells were then fixed using 3.7% paraformaldehyde for 30 min followed by mounting on slides using DABCO (1,4-diazbicyclo (2,2,2) octane (55) 2.5% in 80% glycerol. (Babuta et al., 2015). Fluorescence images were obtained, at 60X magnification using GFP filter set at 488 nm excitation and 510 nm emission in an Olympus Fluoview FV1000 laser scanning microscope.

### Phagocytosis of Bacterial Population Isolated From Whole Stool Sample of Healthy Volunteers

Whole stool sample was collected from two healthy age and sex matched volunteers, who were not suffering from any gastrointestinal disease and did not consume any antibiotics for 6–8 weeks prior to sample collection. Each volunteer gave informed consent for the study. About 200 mg of fresh stool sample was taken in 1 ml of sterile PBS containing 0.1% cysteine (PBSC), homogenized by vortexing for 5 min. Suspension was allowed to stand at room temperature (RT) for 5 min to allow large solid particles to settle down to the bottom of the tube. The vial was centrifuged at 1,200 × g for 5 min, and the supernatant containing all the bacteria harvested from stool was taken into a sterile tube, centrifuged at 14,000 × g and the supernatant was discarded. The pellet was then washed with PBSC and resuspended in 200 μl PBSC and kept at 37°C. The bacteria were pelleted again just before incubation with amoeba for the phagocytosis assay.

The bacterial pellet obtained from whole stool was incubated with *E. histolytica* trophozoites at the ratio of 1:1,000. (Amoeba: Bacteria) in incomplete TYI-S-33 medium in 1.5 ml Eppendorf tubes and incubated at 37°C for 15 min to allow phagocytosis of the bacteria to occur. A negative control with bacterial pellet from one stool sample was also incubated at 4°C for 15 min to inhibit phagocytosis. The amoebae were then washed with ice cold PBSC containing 5 mM Na azide and 50 μM gentamycin and centrifuged at 300 × g for 5 min at 25°C. This process was carried out 2–3 times to remove all the surplus bacteria. The pellet containing amoebic cells were finally lysed and DNA was isolated using QIAamp Fast DNA stool mini kit. The bacterial species phagocytosed by the amoeba were identified by 16S rRNA metagenomic analysis (V1-V5).

### Metagenomic Analysis

**Figure 1:**
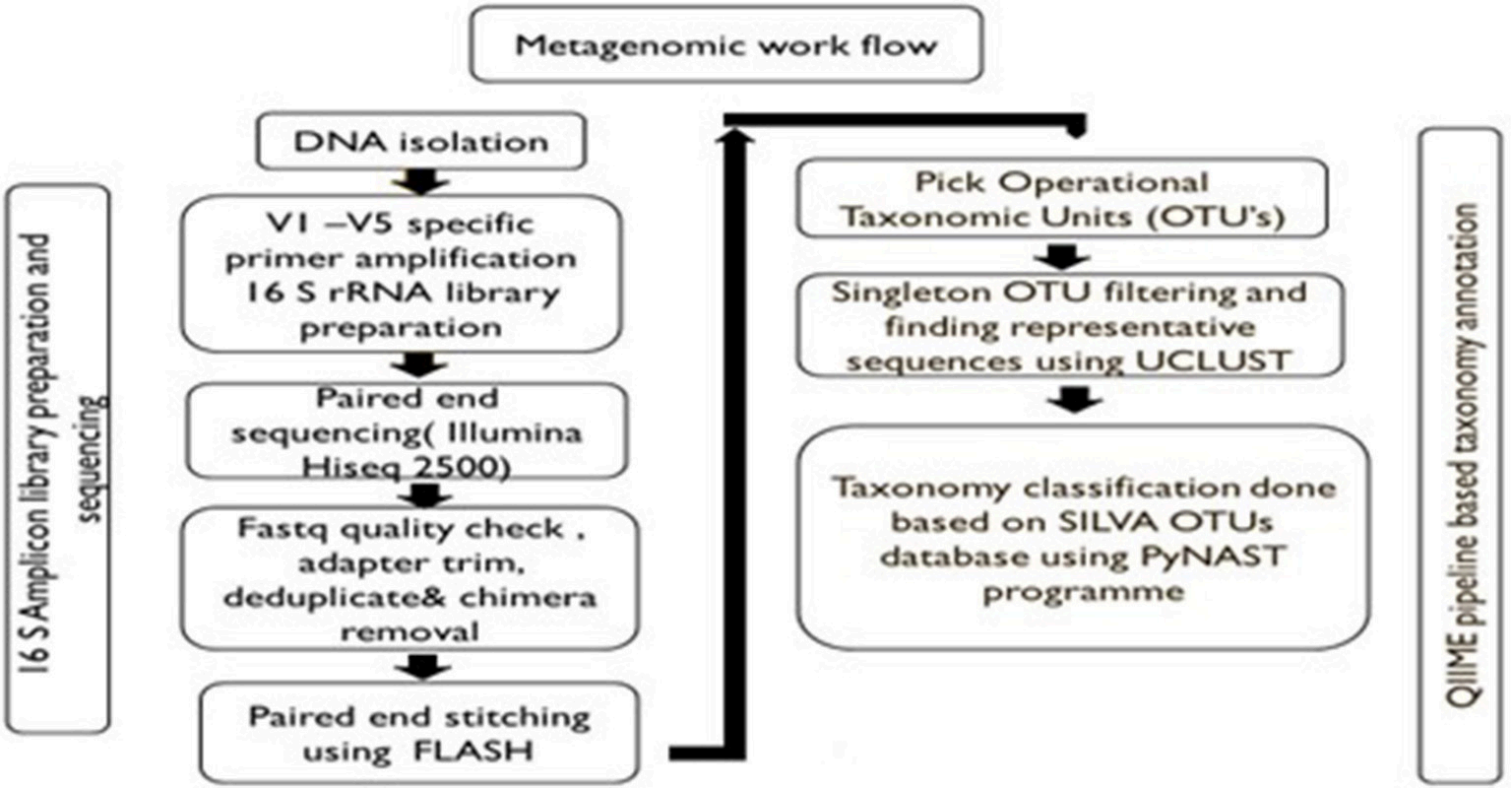
Pipeline for metagenomics analysis.

Amplification and sequencing of the 16S rDNA V1-V3 & V3-V5 regions was done using Illumina HiSeq 2500 Rapid/MiSeq sequencing platforms using the DNA of bacterial groups phagocytosed by amoeba and the DNA from the whole stool as control. Universal bacterial primers were used for PCR amplification. PCR reaction volume of 25 μl was set up using NEB Taq polymerase. PCR conditions were as follows: initial denaturation at 95°C for 30 s, followed by 30 cycles of 95°C for 30 s; annealing at specific temperatures for 45 s; and 68°C for 30 s. The final extension temperature was 72°C for 5 min. Primer compositions and annealing temperatures are given in Table 1. The PCR products were purified using Pure link kit and were used to build a library for sequencing on Illumina platforms. Libraries from each of the sample were tracked using a sequence barcode. Library quantification was done on Qubit 3.0. Short read sequences obtained from the NGS platform were analyzed by a standard pipeline for metagenomics analysis shown as a flow chart in Figure 1. Briefly, V1-V5 regions from Illumina paired-end sequences were extracted, read quality was checked followed by trimming of spacer and conserved regions. The paired end sequence length was 250 bp. The consensus V1-V5 region sequence is constructed using FLASH program. Filters such as conserved region filter and mismatch filter were performed to take further only the high quality V1-V5 region sequences for various downstream analyses. Chimera were also removed. The average Phred score was >30 and average GC content >50.

**Table 1:** Primer composition and annealing temperatures for the PCR amplifications.

**Primer position**	**Composition of primer pairs**	**Annealing temperature**
V1-V3 Outer	F_AGRGTTYGATYMTGGCTCAG _ATTACCGCGGCTGCTGGC	61°C
V1-V3 Inner	F_GCATGCCTAACACATGCAA R_GCTGCTGGCACGGAGTTAG	63°C
V3-V5 Outer	F_CCTACGGGAGGCAGCAG R_CCGTCAATTCMTTTRAGT	58°C
V3-V5 Inner	F_AAGGCCTTCGGGTTGTAA R_CCTTTGAGTTTCAGCCTTGC	62°C

OTUs, Taxonomy classification and relative abundance analysis were performed using the pre-processed consensus V1-V5 sequences in QIIME. Pre-processed reads from all samples were pooled and clustered into Operational Taxonomic Units (OTUs) based on their sequence similarity using UCLUST algorithm (similarity cutoff = 0.97). Representative sequence was identified for each OTU and aligned against Greengenes core set of sequences using PyNAST program. Taxonomy classification was performed using RDP classifier against SILVA OTUs database. The phylum, class, order, family, genus, and species distribution for each sample based on OTU and reads were obtained. The taxa other than top 10 were categorized as others. The sequences not showing any alignment against taxonomic database were categorized as unknown.

## Results

### Uptake of Bacteria by *E. histolytica* Cells During Phagocytosis

**Figure 2:**
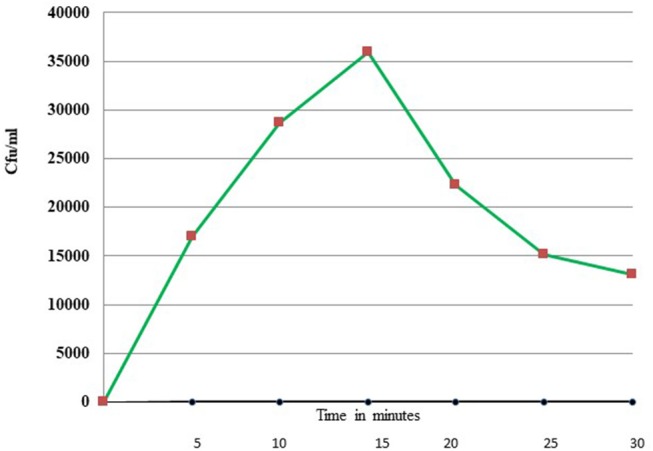
Phagocytosis of *E. coli* (O55) by *E. histolytica* (HM-1:IMSS) at different time points measured by colony forming units.

**Figure 3:**
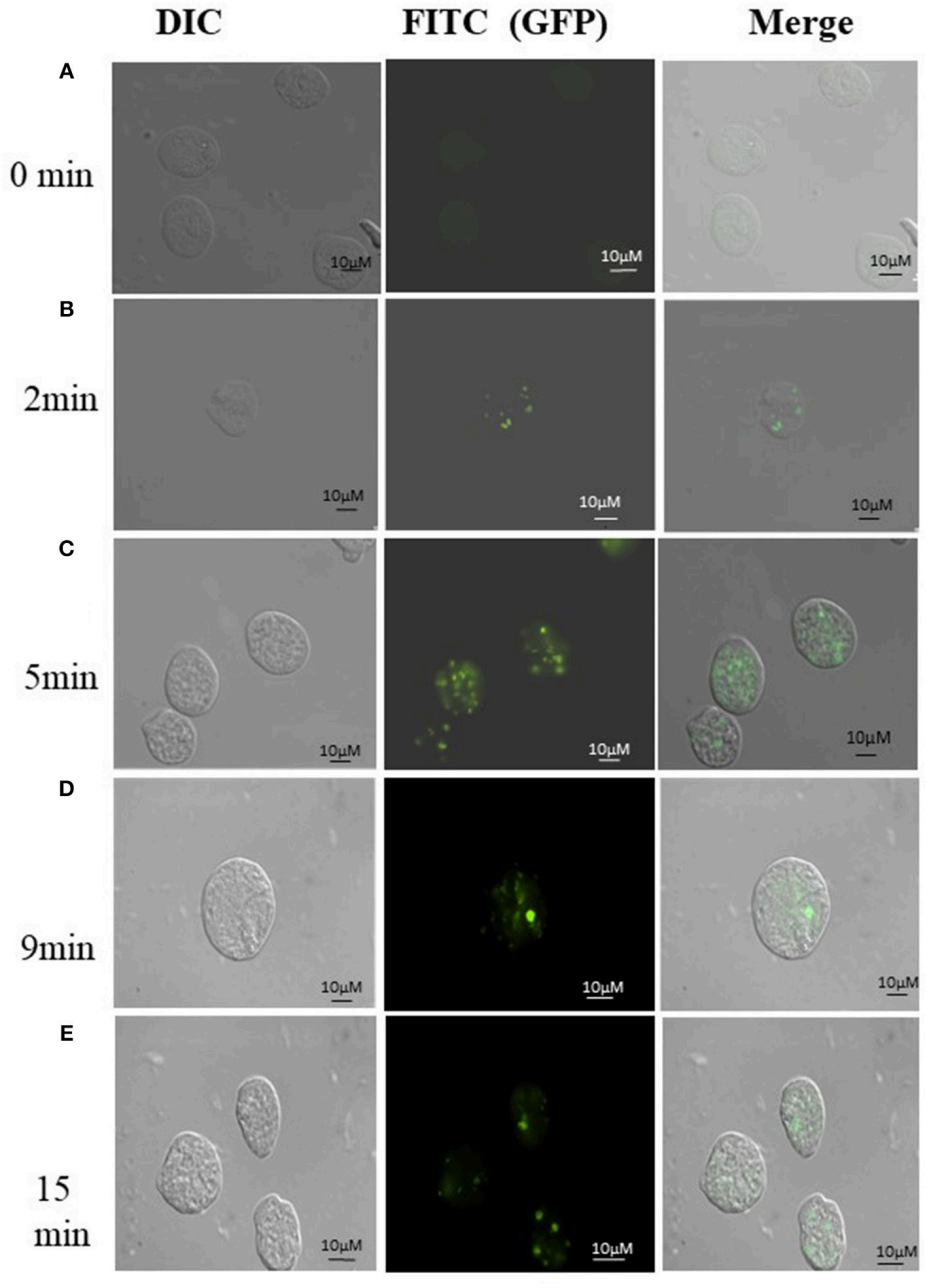
**(A-E)** Phagocytosis of *E. coli* (C41-DE3) carrying a GFP tag, by *E. histolytica* HM-I:IMSS at time points 0, 2, 5, 9, and 15 min. The images from left to right in each panel show DIC (gray), immunolocalization of GFP tagged E.coli using FITC, and the merged image. Scale bar: 10 μm.

Uptake of bacteria by *E. histolytica* was initially studied using *E.coli* (O55) strain. Figure 2 shows the number of colony forming units (CFUs) obtained at indicated times after incubation of *E.coli* with *E. histolytica*. The results showed that maximum bacterial uptake took place at 15 min and on further incubation the number of CFUs decreased. There was no colony formation in the negative control when bacteria were incubated with the parasite at 4°C. Therefore, further bacterial uptake studies were carried out at 37°C for a time period of 15 min. Fluorescence microscopic examination of amoebic cells after incubation with GFP tagged *E. coli*, C41-DE3 showed the phagocytosis of GFP-tagged bacteria by *E. histolytica* cells in Figures 3A–E from a time period of 0–15 min It is seen from the images that initially no *E.coli* cells are seen inside the parasite and later *E. coli* cells were engulfed by the parasite. It was also seen that there were no GFP tagged *E. coli* cells adhering to the surface of the amoeba suggesting that using the above protocol, no non-phagocytosed bacteria remained in the preparation after washing.

### Metagenomics Analysis

Bacteria engulfed by *E. histolytica* cells from a preparation of fecal material were identified by rRNA based metagenomic pipeline as described in Methodology. In both the replicate sets, a sequence library consisting of 250 × 2 base pairs was created. The raw read summary suggested a GC content of >50% and a Phred quality score >35. The read summary of the V1-V5 paired end sequences after trimming is shown in the Table 2.

**Table 2:** Read Summary of V1-V5 paired end sequences.

	**Sample ID**	**Total reads**	**Reads passed filter**
**SET-1**
V1-V3	PP3715-1	481,153	475,494
	WS-1	472,921	468,773
V3-V5	PP3715-1	533,071	529,192
	WS-1	694,790	684,819
**SET-2**
V1-V3	PP3715-2	699,466	606,820
	WS-2	813,721	553,909
V3-V5	PP3715-2	1,057,857	1,037,381
	WS-2	2,521,460	2,464.614

### OTUs, Taxonomy Classification and Relative Abundance Analysis

All samples were pooled and clustered into Operational Taxonomic Units (OTUs) based on their sequence similarity (similarity cutoff = 0.97). Table 3 shows the number of OTUs selected for further analysis in each set.

**Table 3:** Summary of Singleton OTUs.

	**Set-1**	**Set-2**
Total Reads	1,604,015	1,684,724
Total OTUs picked	1,018,761	659,733
Total Singleton OTUs	907,654	554,112
Total OTUs after singleton removal	111,107	105,621

#### Metagenomic Analysis of Phagocytosed Microbiota by the Parasite

**Figure 4:**
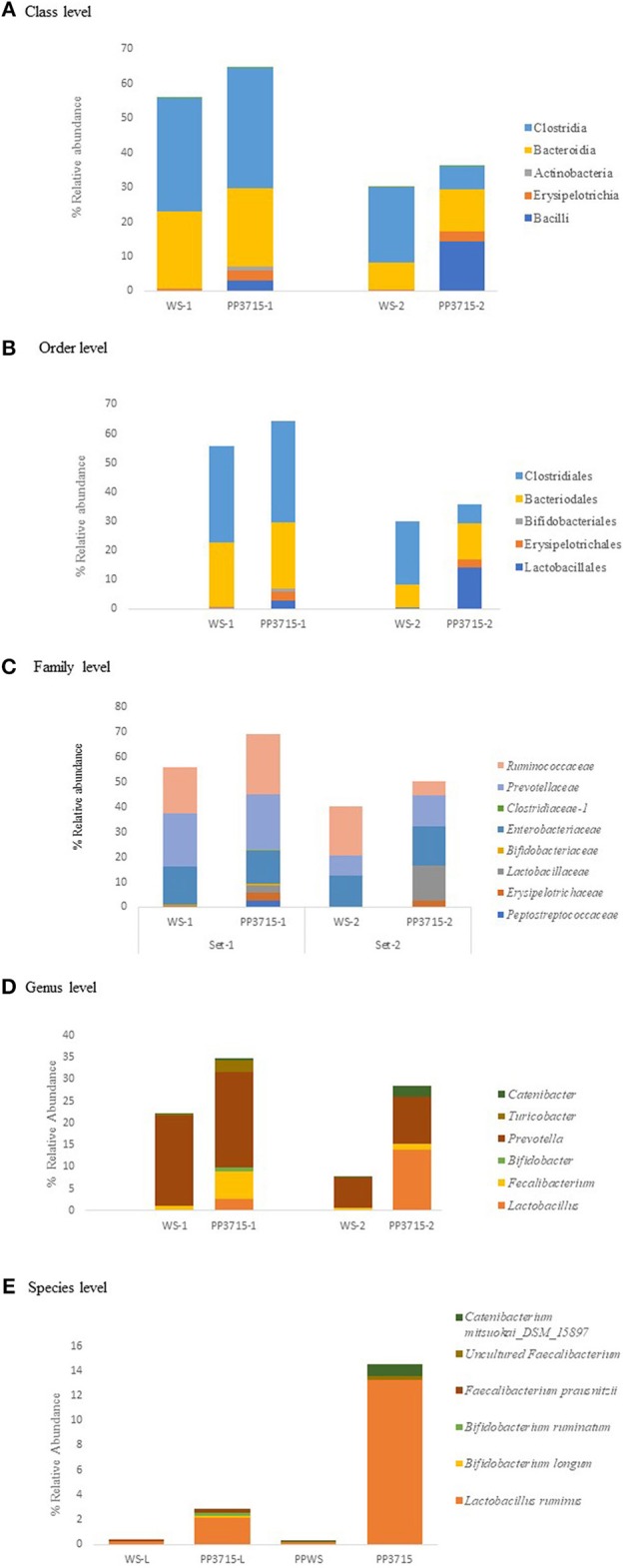
show the bacteria phagocytosed by *E. histolytica* at the class, order, family, genus, and species levels in replicates Set-1 and Set-2 respectively. Prefix (WS) represents the starting bacterial population (control) and prefix (PP) represents the phagocytosed bacteria in each set. 3715 represents incubation at 37°C for 15 min.

The results of analysis of metagenomic sequencing data of bacterial population phagocytosed by *E. histolytica* (HM-1: IMSS) cells in replicate sets 1 and 2, respectively, after a period of 15 min incubation at 37°C are shown in Table 4. The bacterial population preferentially phagocytosed was obtained by comparing the percent relative abundance of bacterial OTUs between the control and the phagocytosed samples. The abundance of bacterial population in the control stool samples were represented by (WS-1) and (WS-2) in Set-1 and Set-2, respectively. The bacterial population phagocytosed in both the sets are represented as (PP3715-1) and (PP3715-2) in Set-1 and Set-2, respectively Enrichment of a bacterial group shown by increase in percent abundance of specific bacterial population in the phagocytosed sample in comparison to control indicate preferential phagocytosis. Figures 4A–E show the percent relative abundance of major bacterial OTUs that showed enrichment at the class, order, family, genus, and species levels, respectively. Table 4 summarizes the bacterial groups preferentially phagocytosed from the class to the species level in the two sets. As can be seen in the table there is a significant fold increase in the percent relative abundance of these bacterial groups in the phagocytosed samples when compared to their respective controls. The bacterial composition of the control stool samples were different however the bacterial groups enriched in the phagocytosed samples were common in both the experiment sets.

**Table 4:** Bacterial groups preferentially phagocytosed by *E. histolytica* in the two replicate sets.

**Level**	**Set-1:Percent relative abundance of bacterial groups**			**Set-2:Percent relative abundance of bacterial groups**		
**Phylum**	**Control (WS-1)**	**PP3715-1**	**Fold increase in % relative abundance**	**Control (WS-2)**	**PP3715-2**	**Fold increase in %Relative abundance**
**CLASS**						
Bacilli	0.1	2.92	29.2	0.22	14.4	65
Erysipelotrichia	0.48	3.14	6.54	0.06	2.66	44
Actinobacteria	0.17	1.14	6.7	0.02	0.09	4.5
Clostridia	32.8	34.7	1.05			
Bacteroidia	–	–	–	8.0	12.31	1.53
**ORDER**						
Lactobacillales	0.10	2.90	29	0.21	14.14	67.3
Erysipelotrichales	0.48	3.14	6.5	0.06	2.66	44.33
Bifidobacteriales	0.05	1.01	20.2	0.01	0.08	8
Clostridales	32.8	34.7	1.05	21.57	6.56	–
Bacteroidales	22.22	22.56	–	8.0	12.31	1.53
**FAMILY**						
*Lactobacillaceae*	0.09	2.62	29.11	0.21	13.99	66.6
*Erysipelotrichaeceae*	0.48	3.14	6.54	0.06	2.66	44.33
*Clostridiaceae-1*	0.015	0.65	43.33	0.003	0.0017	0.56
*Prevotellaceae*	20.96	22.19	1.05	7.96	12.26	1.54
*Peptostreptococcaceae*	0.15	2.69	17.93	–	–	
*Ruminococcaceae*	18.46	24.15	1.30	19.89	5.61	–
*Bifidobacteriaceae*	0.05	1.01	20.2	0.011	0.082	7.45
*Enterobacteriaceae*	15.52	12.60	–	12.26	15.81	1.28
**GENUS**						
*Lactobacillus*	0.09	2.62	29.11	0.21	13.99	66.61
*Bifidobacterium*	0.042	0.827	19.69	0.011	0.080	7.27
*Faecalibacterium*	1.03	6.33	6.14	0.45	1.15	2.55
*Prevotella*	20.59	21.77	1.05	7.06	10.82	1.53
*Turicobacter*	0.27	2.85	10.55			
*Catenibacter*	0.188	0.198	1.05	0.009	2.38	264.44
**SPECIES**						
*Lactobacillus ruminus*	0.24	2.15	8.95	0.19	13.25	69.73
*Faecalibacterium prausnitzii*	0.15	0.35	2.33			
*Bifidobacterium ruminantum*	0.01	0.21	21			
*Bifidobacterium longum*	0.015	0.194	12.93			
*Uncultured Faecalibacterium*				0.11	0.34	3.09
*Catenibacterium mitusuokai DSM 15897*	–	–	–	0.0039	0.9426	241.69

At the class level, (Figure 4A, Table 4), Bacilli, Erysipelotrichia, and Actinobacteria were preferentially phagocytosed in both the replicates and showed significant fold increases. Bacteroidia showed fold increase in Set-2. Members of Lactobacillales, Erysipelotrichales and Bifidobacteriales at the order level were enriched in both sets, Bacteroidales showed fold increase in Set-2 (Figure 4B, Table 4). At the family level, bacteria belonging to *Lactobacillaceae, Erysipelotrichaeceae, Clostridiaceae-1, and Bifidobacteriaceae* were selectively taken up in Set-1 and Set-2. *Peptostreptococcaceae* and *Ruminococcaceae* was taken up in Set-1, while *Prevotellaceae* and *Enterobacteriaceae* was taken up in Set-2 (Figure 4C, Table 4). At the Genus level, bacteria belonging to *Lactobacillus, Faecalibacterium and Bifidobacterium* were the predominant genera enriched in both the sets. (Figure 4D, Table 4). Genus *Turicobacter* was also enriched in Set-1 and Genus *Prevotella* and *Catenibacter* were enriched in Set-2. *Lactobacillus ruminus* was the main species that showed significant enrichment consistently in both the sets. *Faecalibacterium prausnitzii* also was enriched in Set-1, while *Catenibacterium mitsuokai* and an uncultured *Faecalibacterium* showed fold increase in Set-2. Other species that showed fold increase in Set 1 were *Bifidobacterium longum* and *Bifodobacterium ruminantium* Figure 4E, Table 4. In a negative control incubated at 4°C in Set-2, the percent relative abundance of *Lactobacillus ruminus* and uncultured *Faecalibacterium is negligible* (Data not shown).

## Discussion

It has long been recognized that gut bacterial flora plays an important role in the biology of the protist parasite *E. histolytica* (Mirelman, 1987). However, it is not clear that if the parasite has specific interaction with only a subset of bacteria present in the gut. If this is true then the biology of amoeba gets modulated by different gut microbiome composition. We have investigated the relationship of amoeba and bacteria by characterizing the bacterial population phagocytosed by *E. histolytica* in comparison to the total gut microbiome population. In order to show specific interaction, we have estimated enrichment of a group or specific organism after phagocytosis. Our results do show enrichment of groups of organisms in phagocytosed population compared to total population. Though the starting bacterial population in the controls were very different our results suggest that the bacteria being enriched in both sets were similar. In both the sets Bacilli, Erysipelotrichia and Actinobacteria are the major class of bacteria being phagocytosed by the parasite. Further, members of the order Lactobacillales of Bacilli class, Erysipelotrichales of Erysipelotrichia class, and Bifidobacteriales of Actinobacteria class are taken up by *E.histolytica*. At the family level we observed that bacteria belonging to the family *Lactobacillaceae, Clostridiaceae, Erysipelotrichaceae, and Bifidobacteriaceae* dominated among the phagocytosed bacteria in both the sets of experiments. Our sequence analysis at the genus level revealed predominance of *Lactobacillus* members followed by members of *Bifidobacterium* and *Faecalibacterium* being preferentially phagocytosed. *Catenibacterium* also showed enrichment in Set-2 This was further confirmed at species level, where we identified species *Lactobacillus ruminus* in both the sets as the species being mainly phagocytosed by the parasite. *Faecalibacterium prausnitzii, Bifidobacterium longum*, and *Bifodobacterium ruminantium were also enriched* in set-1 and *Catenibacterium mitsuokai* was enriched in Set-2. In a negative control run with experiment set-2, incubated at 4°C to inhibit phagocytosis, relative abundance of *Lactobacillus ruminus* was negligible in the sequence analysis at the species level.This evidence suggests that this organism is taken up by the parasite and is not attached to the surface of *Entamoeba* trophozoites. The preferential phagocytosis of *L. ruminus* in both experiment sets is a novel observation as this species has never been reported to be in association with *E. histolytica*.

Our findings reveal that the members of bacteria phagocytosed by *Entamoeba* constitute the healthy gut flora. It has been demonstrated earlier that the members belonging to the order *Lactobacillales* perform many important functions in the gut, such as production of Bacteriocins to supress the growth of pathogenic bacteria, synthesis of lactic acid and H_2_O_2_, fortification of epithelial barrier by induction of mucin secretion and enhancement of tight-junction functioning (Lebeer et al., 2008) and help in the prevention of infectious diarrhea, antibiotic associated diarrhea and diarrhea in children (Van Neil et al., 2002). Varet et al. (2018) have shown that the gene responses observed in *E. histolytica* in the presence of Enterobacteriaceae favoring their survival in the presence of the oxidative stress do not occur during coculture with *L. acidophilus*.

*Lactobacillus ruminus*, the major bacterial species phagocytosed by *E. histolytica* as observed here, is an autochthonous member of healthy gut and considered to be a potential probiotic. It can produce flagella (O'Donnell et al., 2015) and inhibits the growth of antibiotic resistant pathogens like vancomycin resistant *Staphylococcus aureus* and *Enterococci*. (Yun et al., 2005). Depletion of *L. ruminus* may promote colonization of the pathogenic bacteria in the gut. However further studies are needed in order to understand why *L. ruminus* is preferentially phagocytosed by *E.histolytica*.

Members of the family *Erysipelotrichaceae* and *Clostridiaceae* were found to be enriched in our phagocytosis experiment. Bacteria belonging to *Erysipelotrichales* are highly immunogenic and play a role in preventing gut inflammation (Kaakoush, 2015). Higher levels of *Catenibacterium* genus, a polysaccharide degrading genus belonging to *Erysipelotrichales* are found in mediterranean diets which are in turn associated with reduced inflammation markers (Shankar et al., 2017). Phagocytosis of *Catenibacterium* by *E. histolytica* could lead to lower levels of beneficial *Catenibacterium* in the gut.

Members of *Clostridiaceae* family are known to produce butyrate as the end product of their fermentation. Butyrates are the preferred energy source for colonocytes and are important anti-inflammatory modulators (Lopetuso et al., 2013). Enrichment of *Bifidobacterium ruminantum* and *Bifidobacterium longum* at the species level suggest their phagocytosis by *E. histolytica*. *B.longum* protect the gut from enteropathogenic infection through the production of acetate (SCFA). Acetate is the major energy source for colonocytes. (Fukuda et al., 2011). Verma et al. (2012) have found significant increase of *Bifidobacterium* species in amoebic patients by Real time PCR. They suggest a cross talk between the intestinal epithelial cells, the bacteria and the parasite, leading to increased mucus secretion by the intestinal cells and colonization of the intestinal epithelium by *Bifidobacterium* species during *E. histolytica* infections.

In conclusion our study shows *E. histolytica* preferentially phagocytosed some of the beneficial bacteria that are required for the maintenance of a healthy gut amongst which are family *Lactobacillales, Erysipelotrichales, Clostridales, and Bifidobacteriales*. Phagocytosis of these bacteria may cause dysbiosis of gut bacteria and creates conditions for the proliferation of the parasite in the human intestinal lumen. At the species level our data shows that preferential phagocytosis of *Lactobacillus ruminus* by Entamoeba was quite significant.

## Data Availability

Raw reads data for this project were deposited at SRA (NCBI) data base with the following accession numbers:

Study: PRJNA515425

1. Sample: PP3715-1 (SAMN10755114)

2. Sample: PP3715-2 (SAMN10755115)

3. Sample: WS-1 (SAMN10755112)

4. Sample: WS-2 (SAMN10755113)

The link to the data is: https://www.ncbi.nlm.nih.gov/Traces/study/?acc=PRJNA515425.

## Ethics Statement

This study was carried out in accordance with the recommendations of IERB-JNU (Institutional Ethics review board of Jawaharlal Nehru University). (IERB Ref. No.2015/Faculty/82) with written informed consent from all subjects. All subjects gave written informed consent in accordance with the Declaration of Helsinki. The protocol was approved by the Institutional Ethics review board of Jawaharlal Nehru University.

## Author Contributions

LI, AV, JP, and AB conceived, designed the experiments, and analyzed the data. LI and AV performed the experiments. JP and AB contributed reagents, materials, and analysis tools. LI, JP, and AB wrote the paper.

### Conflict of Interest Statement

The authors declare that the research was conducted in the absence of any commercial or financial relationships that could be construed as a potential conflict of interest.
